# TMPRSS4 as a Poor Prognostic Factor for Triple-Negative Breast Cancer

**DOI:** 10.3390/ijms140714659

**Published:** 2013-07-12

**Authors:** Daye Cheng, Hong Kong, Yunhui Li

**Affiliations:** 1 Department of Transfusion, The First Hospital of China Medical University, Shenyang 110001, Liaoning, China; 2 Department of Clinical Laboratory, Shengjing Hospital of China Medical University, Shenyang 110004, Liaoning, China; E-Mail: konghong73@163.com; 3 Department of Clinical Laboratory, No. 202 Hospital, Shenyang 110003, Liaoning, China; E-Mail: liyh202@126.com

**Keywords:** transmembrane protease, triple-negative breast cancer, prognosis, immunohistochemistry

## Abstract

Triple-negative breast cancer (TNBC) is characterized by the lack of immunohistochemical staining for estrogen receptors (ER), progesterone receptors (PR), and lack of overexpression or amplification of human epidermal growth factor receptor 2 (HER2). Our aim was to investigate the expression of transmembrane protease, serine 4 (TMPRSS4) in TNBC patients and its possible relationship to the outcome of the disease. A total of 72 TNBC patients and 109 non-TNBC patients who were diagnosed between 2003 and 2008 were enrolled in this study. Immunohistochemistry was used to compare the expression pattern of TMPRSS4 in TNBC and non-TNBC groups, and the prognostic significance was assessed by Kaplan-Meier analysis and Cox proportional hazards regression in TNBC patients. The rate of high expression of TMPRSS4 was significantly higher in TNBC group than that in non-TNBC group. High expression of TMPRSS4 was significantly correlated with lymph node metastasis, histological grade, and tumor size. TNBC patients with high TMPRSS4 expression showed the poorer overall survival (OS) and disease-free survival (DFS) than those patients with low TMPRSS4 expression. In multivariate analysis, only lymph node metastasis and TMPRSS4 expression were the independent prognostic factors for OS and DFS in TNBC. Our study provides evidence that TMPRSS4 expression is associated with lymph node metastasis, tumor size, and histological grade in TNBC patients, and also is an independent prognostic factor for TNBC.

## 1. Introduction

Breast cancer remains the most frequently diagnosed female cancer worldwide and the leading cause of cancer death, despite screening and improvements in adjuvant treatment [1]. Triple-negative breast cancer (TNBC) is a molecular subtype of breast cancer characterized by the lack of immunohistochemical staining for estrogen receptors (ER), progesterone receptors (PR), and lack of overexpression or amplification of human epidermal growth factor receptor 2 (HER2) [2–4]. Due to high rates of visceral and central nervous system metastases, patients with TNBC have a poorer disease specific survival than hormone receptor-positive subtypes [5–7]. However, predictive and prognostic factors of TNBC phenotype are poorly understood. Therefore, there is an urgent need to identify new prognostic biomarkers that can be used to predict a therapeutic response and clinical outcomes in TNBC patients to rationalize treatment decisions.

Recently, type II transmembrane serine proteases (TTSPs) have been recognized as a new subfamily of serine proteases, which are implicated in tumor development and progression [8–10]. Transmembrane protease, serine 4 (TMPRSS4), as a member of TTSPs, was first discovered by differential gene analysis for pancreatic cancer markers [11]. To date, several studies have reported high expression at the transcriptional level in colorectal, thyroid, lung, and pancreatic cancer by Northern blot analyses, microarray gene chips, and RT-PCR. Further analysis of TMPRSS4-mediated signaling in colon cancer cells suggested that multiple downstream signaling pathways are activated including focal adhesion kinase (FAK) and extracellular signal regulated kinase (ERK) resulting in the downregulation of *E*-cadherin and induced expression of integrin α5, a critical molecule implicated in tumor cell invasion, migration and tumor progression [12,13]. In addition, Jung H, *et al.* have reported that elevated TMPRSS4 expression induced epithelial to mesenchymal transition (EMT) of cancer cells and promoted metastasis [14].

Recently, we found that TMPRSS4 was overexpressed in breast cancer tissue, but few studies document the expression of TMPRSS4 in TNBC and non-TNBC, especially in TNBC. In this study, we investigated the expression of TMPRSS4 in TNBC and non-TNBC by immunohistochemistry and evaluated the prognostic value of TMPRSS4 for overall survival (OS) and disease free survival (DFS) in TNBC patients.

## 2. Results and Discussion

### 2.1. The Expression Profiles of TMPRSS4 in Breast Cancer Tissues

**Figure 1:**
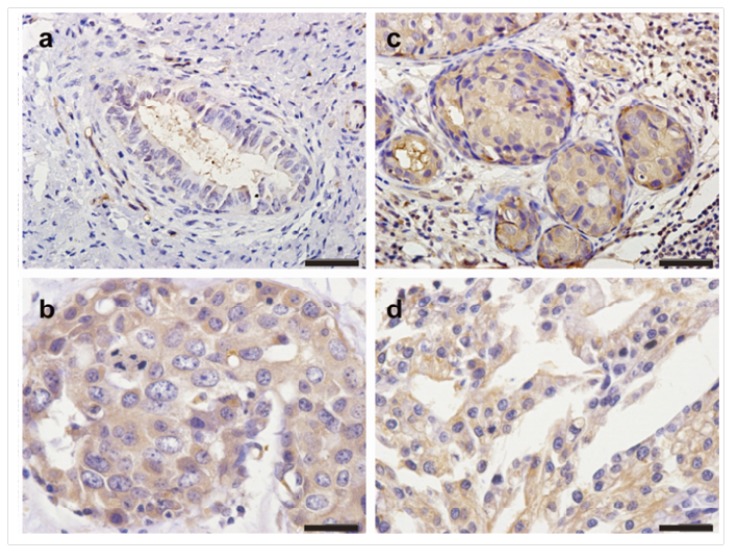
Immunohistochemical detection of TMPRSS4 expression in formalin-fixed paraffin-embedded triple-negative breast cancer (TNBC) tissue samples. (**a**) Negative TMPRSS4 immunostaining in another TNBC tissue; (**b**–**d**) Positive TMPRSS4 immunostaining in TNBC tissue. Positive TMPRSS4 immunostaining in TNBC tissues appeared as brown particles, which were mainly localized within the cytoplasm and cell membrane in the nests of breast cancers.

The clinical characteristics of patients were shown in Table 1. In all 181 breast cancer patients, high expression of TMPRSS4 was observed in 62.4% of breast cancer patients. Immunohistochemical examination showed that TMPRSS4 was located in the cytoplasm and cell membrane in nests of tumor cells. We also observed that some stromal fibroblasts, peripheral nerves, and vascular smooth muscle cells showed a weak and sparse immunoreactivity, but not specific. The different intensities of the staining were shown in Figure 1. The rate of high expression of TMPRSS4 was significantly higher in TNBC group (73.6%) than that in non-TNBC group (55.0%) (*p* = 0.012). Comparison of clinical features between TNBC group and non-TNBC group showed that histological subtype (*p* = 0.003), lymph node metastasis (*p* = 0.004) and tumor size (*p* = 0.048) were significantly different.

To date, many studies demonstrate that TTSPs participate in the regulation of cellular signaling events at the plasma membrane and in the extracellular matrix [8]. Many of the TTSPs show restricted tissue distribution in normal cells, but their expression is increased during the tumor growth and progression [15]. TMPRSS4, as an important member of TTSPs, is reported to involve in invasion, metastasis, migration and adhesion, as well as in the mesenchymal-epithelial transition (EMT) in cancer cells and it also modulates cell growth in a cell type-dependent manner [14]. Moreover, many reports suggested that TMPRSS4 is associated with tumor progression. Larzabal *et al.* have revealed that knockdown of TMPRSS4 by shRNA reduces significantly cell migration *in vitro* in H358, H441 and H2170 cell lines, thus implying a role for TMPRSS4 in metastasis [13,16]. Recent qPCR studies showed that high levels of TMPRSS4 message in NSCLC patients were associated with a poor prognosis [16]. In the present study, we showed that the rate of high TMPRSS4 expression was significantly higher in TNBC group than that in non-TNBC group.

### 2.2. Correlation of TMPRSS4 Expression in TNBC with Clinicopathological Characteristics

The associations between TMPRSS4 expression and a series of clinicopathological factors were evaluated in TNBC group. As shown in Table 2, high expression of TMPRSS4 was significantly correlated with lymph node metastasis (*p* = 0.005), histological grade (*p* = 0.033), and tumor size > 2 cm (*p* = 0.007), but not correlated with other clinicopathological parameters, including the patient’s age (*p* = 0.737), menopausal status (*p* = 0.188), and histological subtype (*p* = 0.378).

TNBC is characterized by a significantly higher probability of relapse and poorer overall survival in the first 3–5 years compared with other breast cancer subtypes [17,18]. The prognostic value of classical pathological variables, such as tumor grade, lymph node status, and tumor size, could be impaired in TNBC. Therefore, there is an urgent need to understand the molecular background of TNBC to delineate the patients with especially poor prognosis and to develop effective treatment for these patients. High TMPRSS4 expression in TNBC specimens was associated with the tumor grade, lymph node metastasis, and tumor size. Overexpression of a cell surface protease has the potential to affect the extracellular matrix and to alter cell morphology thereby enhancing cell motility and invasiveness of distant organs. Kim *et al.* demonstrated that overexpression of TMPRSS4 enzyme leads to the breakdown of extracellular matrix, and promotes invasion and migration of cancer cells in cell-based assays and induced the expression of integrin α5 [12]. Moreover, tail vein injection of H358 tumor cells knocked-down for expression of TMPRSS4 with shRNA resulted in decreased tumor metastasis to the lung [16]. Our findings reinforce the main role of TMPRSS4 in cancer development.

### 2.3. Survival Analysis Correlation of TMPRSS4 Expression in TNBC with Clinicopathological Characteristics

**Figure 2:**
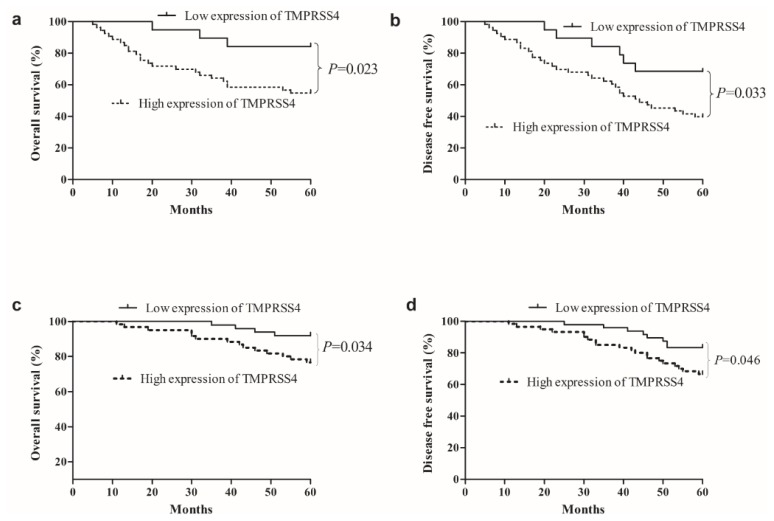
Kaplan-Meier curves for overall survival (OS) and disease-free survival (DFS) of TNBC patients stratified by TMPRSS4 expression. (**a**) OS curves of TNBC patients according to TMPRSS4 immunostaining; (**b**) DFS curves of TNBC patients according to TMPRSS4 immunostaining; (**c**) OS curves of non-TNBC patients according to TMPRSS4 immunostaining; (**d**) DFS curves of non-TNBC patients according to TMPRSS4 immunostaining. *p-*Values were obtained by log-rank test.

The OS and DFS of TNBC and non-TNBC patients based on TMPRSS4 expression were shown in Figure 2. Notably, high expression of TMPRSS4 was significantly associated with a reduced 5-year survival for TNBC patients and non-TNBC patients (TBNC group: OS: *p* = 0.023; DFS: *p* = 0.033, Non-TNBC group: OS: *p* = 0.034; DFS: *p* = 0.046, respectively). The 5-year OS was 54.7% in TNBC patients displaying high TMPRSS4 expression, while it was 84.2% in TNBC patients displaying low TMPRSS4 expression. Similarly, the 5-year DFS was 39.6% in TNBC patients displaying high TMPRSS4 expression, while it was 68.4% in TNBC patients displaying low TMPRSS4 expression. These data suggest that high TMPRSS4 expression is a poor prognostic indicator for TNBC patients.

In Table 3, univariate analysis revealed that lymph node metastasis (OS, *p* = 0.012; DFS, *p* = 0.018), tumor size (OS, *p* = 0.046; DFS, *p* = 0.049), grade (OS, *p* = 0.029; DFS, *p* = 0.044), and TMPRSS4 expression (OS, *p* = 0.032; DFS, *p* = 0.040) were prognostic factors in TNBC patients for OS and DFS, respectively. In multivariate analysis, only lymph node metastasis (OS, *p* = 0.020; DFS, *p* = 0.026) and TMPRSS4 expression OS, *p* = 0.037; DFS, *p* = 0.045) were the independent prognostic factors for OS and DFS in TNBC. In summary, high TMPRSS4 expression was associated with OS and DFS in TNBC, and a potential prognostic indicator for TNBC patients.

Poor prognosis was evidenced by low DFS and OS in patients with high TMPRSS4 expression. Multivariate analysis indicated that TMPRSS4 was an independent prognostic factor for the OS and DFS. These findings suggest that elevated expression of TMPRSS4 contributes to the aggressive phenotype in TNBC patients. More notably, high expression of TMPRSS4 might be able to predict a worse prognosis in TNBC patients. Furthermore, these results have important implications for the design of novel therapeutic intervention for TNBC patients, especially those who seem to have poor prognosis.

## 3. Experimental Section

### 3.1. Patients

From 2003 to 2008, a total of 181 cases of newly diagnosed and surgically treated breast cancer patients at the Cancer Center of No. 202 Hospital were included in this study. The mean age of the 181 patients was 50.2 years (range, 21–78 years). Patients who met the following eligibility criteria were included: (1) All patients had no previous surgery to the breast and did not receive neoadjuvant chemotherapy or radiation therapy before surgery; (2) Availability of follow-up data; (3) No history of familial malignancy. Histological type was reclassified according to WHO classification, and stage of tumor was based on TNM staging system (American Joint Committee on Cancer Classification) [19]. According to the expression of ER, PR, and HER-2, patients were grouped as TNBC group and non-TNBC group. Clinicopathological features of the patients were collected by the retrospective review of medical archives. All patients were followed up by interview in hospital or phone call. This study was approved by the Ethics Committee of No. 202 Hospital, and informed consent was obtained from each patient.

### 3.2. Immunohistochemical Staining

Immunohistochemical analysis of breast tissue was performed as described previously before. Briefly, paraffin sections were cut at 4 μm thickness, mounted on silane coated slides and incubated overnight at 37 °C. Sections were washed with distilled water after two changes of xylene and three changes of ethanol. Antigen retrieval was performed using citrate buffer (pH 6.0) and sections were held in Tris buffered saline (TBS). Endogenous peroxidase activity was blocked by incubation in 3% hydrogen peroxide. The sections were incubated overnight in primary antibody against TMPRSS4 (Proteintech Group, Inc., Wuhan, China) diluted with 1/50 in 1% BSA in Tris buffer (100 mM, pH 7.6) at room temperature. Antibody binding was amplified using horseradish peroxidase-conjugated goat anti-rabbit IgG for 15 min each and the complex was visualized using DAB Horseradish Peroxidase Color Development Kit ((Maixin Co., Fuzhou, China).

### 3.3. Scoring

After staining, sections were assessed microscopically for positive DAB staining by two observers blinded to the clinical data to ensure consistency. The percentage of positive-staining tumor cells was scored as follows: 0 (no positive tumor cells), 1 (<15% positive tumor cells), 2 (15%–50% positive tumor cells), and 3 (>50% positive tumor cells). In cytoplasm, staining intensity was graded as follows: 0 (no staining); 1 (weak staining); 2 (moderate staining); and 3 (strong staining). The staining intensity score plus the percentage of positive staining was used to define the TMPRSS4 expression levels: 0–2, low expression and 3–6, high expression, which classified breast cancer patients into two groups.

### 3.4. Statistical Analysis

All data were analyzed using SPSS 13.0 software (SPSS, Chicago, IL, USA). Relationship between TMPRSS4 expression and clinical parameters were analyzed using χ^2^ test. OS was calculated from TNBC diagnosis to the date of death for any cause, and DFS was defined as the time from TNBC diagnosis to any event related to breast cancer (local or regional relapse, distant metastasis, contralateral breast cancer, or death, whichever occurred first). The Kaplan-Meier curves were plotted to calculate 5-year survival curves, and log-rank test was used to estimate the differences. Clinicopathologic factors known to be associated with prognosis were tested in univariate analysis. Variables that were found to be significant in univariate analysis were then entered in a multivariate Cox proportional hazards regression model to identify those with independent prognostic information for DFS and OS. A *p* < 0.05 was defined as statistically significant.

## 4. Conclusions

In conclusion, our study provides evidence that TMPRSS4 expression is associated with lymph node metastasis, tumor size, and histological grade in TNBC patients, and also is an independent prognostic factor for TNBC. Nevertheless, the potential mechanism between increased TMPRSS4 expression and cancer progression in TNBC is still unknown. Further studies and more samples will be required to investigate the prognostic role of TMPRSS4 in TNBC. Based on these data, we propose that TMPRSS4 may be an attractive and promising therapeutic target for breast cancer patients, especially in TNBC patients.

## Figures and Tables

**Table 1:** Clinical characteristics of breast cancer patients.

Parameters	Total	TNBC (*n* = 72)	Non-TNBC (*n* = 109)	*p-*value
Age (years)				
≤50	67	28 (38.9%)	39 (35.8%)	0.6715
>50	114	44 (61.1%)	70 (64.2%)	

Menopausal status				
Premenopausal	93	40 (55.6%)	53 (48.6%)	0.3611
Postmenopausal	88	32 (44.4%)	56 (51.4%)	

Histological subtype				
Ductal	157	69 (95.8%)	88 (80.7%)	0.003
Lobular	24	3 (4.2%)	21 (19.3%)	

LN metastasis				
Negative	135	41 (56.9%)	84 (77.1%)	0.004
Positive	46	31 (43.1%)	25 (22.9%)	

Tumor size				
≤2 cm	63	27 (37.5%)	26 (23.9%)	0.048
>2 cm	118	45 (62.5%)	83 (76.1%)	

Grade				
I, II	100	38 (52.8%)	62 (56.9%)	0.5969
III	81	34 (47.1%)	47 (43.1%)	

TMPRSS4				
Low expression	68	19 (26.4%)	49 (45.0%)	0.012
High expression	113	53 (73.6%)	60 (55.0%)	

Values are presented as number (%); TNBC = Triple-negative breast cancer.

**Table 2:** Correlation between TMPRSS4 expression and clinicopathological parameters in TNBC patients.

Parameters	Low TMPRSS4 expression (*n* = 19)	High TMPRSS4 expression (*n* = 53)	χ^2^ value	*p*-value
Age (years)				
≤50	8 (42.1%)	20 (37.7%)	0.112	0.737
>50	11 (57.9%)	33 (62.3%)		

Menopausal status				
Premenopausal	13 (68.4%)	27 (50.9%)	1.730	0.188
Postmenopausal	6 (31.6%)	26 (49.1%)		

Histological subtype				
Ductal	18 (94.7%)	51 (96.2%)	0.078	0.378
Lobular	1 (5.3%)	2 (3.8%)		

LN metastasis				
Negative	16 (84.2%)	25 (47.2%)	7.827	0.005
Positive	3 (15.8%)	28 (52.8%)		

Tumor size				
≤2 cm	12 (54.8%)	15 (45.2%)	7.250	0.007
>2 cm	7 (26.9%)	38 (73.1%)		

Grade				
I, II	14 (73.7%)	24 (45.3%)	4.527	0.033
III	5 (26.3%)	29 (54.7%)		

Values are presented as number (%).

**Table 3:** Univariate and multivariate Cox Proportional Hazards Model for OS and DFS in TNBC patients.

Variables	Univariate			Multivariate		
	
	HR	95% CI	*p*-value	HR	95% CI	*p*-value
**OS**						
Age (≤50 *vs.* >50 years)	1.395	0.531–2.856	0.534			
Menopausal status (Pre- *vs.* Post-)	1.112	0.495–2.141	0.707			
Histological subtype (Ductal *vs.* Lobular)	0.937	0.644–1.920	0.832			
Lymph node metastasis (Negative *vs.* Positive)	5.007	2.022–9.637	0.012	4.003	1.985–8.023	0.020
Tumor size (≤2 cm *vs.* >2 cm)	2.811	1.007–5.841	0.046	2.120	1.023–4.111	0.138
Grade (I, II *vs.* III)	3.223	1.021–4.888	0.029	2.744	1.011–4.746	0.109
TMPRSS4 expression (Low *vs.* High)	3.041	1.417–8.036	0.032	3.009	1.419–6.322	0.037

**DFS**						
Age (≤50 *vs.* >50 years)	1.266	0.614–2.563	0.661			
Menopausal status (Pre- *vs.* Post-)	1.104	0.799–2.251	0.721			
Histological subtype (Ductal *vs.* Lobular)	0.916	0.647–1.985	0.840			
Lymph node metastasis (Negative *vs.* Positive)	4.230	1.811–6.829	0.018	3.734	1.666–7.140	0.026
Tumor size (≤2 cm *vs.* >2 cm)	2.763	1.519–6.988	0.049	2.005	1.112–3.814	0.151
Grade (I, II *vs.* III)	2.851	1.003–4.771	0.044	2.612	1.001–4.320	0.085
TMPRSS4 expression (Low *vs.* High)	2.997	1.221–6.470	0.040	2.836	1.302–6.121	0.045

OS, overall survival; DFS, Disease-free survival; HR, Hazard ratio; CI, confidence interval.
